# HiC2Self: Self-supervised denoising for bulk and single-cell Hi-C contact maps

**DOI:** 10.1126/sciadv.adu8060

**Published:** 2026-07-23

**Authors:** Rui Yang, Alireza Karbalayghareh, Christina S. Leslie

**Affiliations:** ^1^ Computational and Systems Biology Program, Memorial Sloan Kettering Cancer Center, New York, NY, USA.; ^2^ Tri-Institutional Program in Computational Biology and Medicine, New York, NY, USA.; ^3^ Broad Institute of MIT and Harvard, Cambridge, MA, USA.; ^4^ Chan Zuckerberg Biohub, New York, NY, USA.

## Abstract

Hi-C is a chromosome conformation capture assay used to study three-dimensional (3D) genome organization. Single-cell Hi-C technologies now enable the examination of 3D chromatin organization in individual cells, although these approaches often suffer from low-coverage libraries and data sparsity. Here, we introduce HiC2Self, a self-supervised framework for denoising Hi-C contact maps that requires only low-coverage data as input. HiC2Self reconstructs key structures such as topologically associating domains (TADs) and significant loops from bulk libraries, including cell-type-specific Hi-C structures, without the generalization challenges faced by supervised models. HiC2Self can also accurately reconstruct significant loops from Micro-C data at 1-kilobase resolution. When applied to single-nucleus methyl-3C data, HiC2Self successfully reconstructs local TAD structures around specific genes at 10-kilobase resolution with as few as 50 cells. Last, HiC2Self enables the examination of single-cell structures at 50-kilobase resolution in individual cells of the same cell type. HiC2Self thus provides a general tool for denoising bulk, pseudobulk, and single-cell 3D contact maps to enable downstream analyses.

## Teaser

HiC2Self is a versatile self-supervised deep learning tool for denoising bulk, pseudobulk, and single-cell Hi-C contact maps.

## INTRODUCTION

Hi-C is a genome-wide chromosome conformation capture assay coupled to next-generation sequencing that is used to study three-dimensional (3D) genomic organization. Hi-C experiments generate paired-end sequencing data that is used to construct a contact matrix between genomic bins. Intrachromosomal Hi-C contact maps can be visualized as symmetric heatmaps, with the *x* and *y* coordinates representing genomic locations along the chromosome. Each pixel in the heatmap shows the intensity of chromatin interaction (normalized read count) between the corresponding bins. Examining Hi-C contact map at various resolutions can reveal different scales of 3D organization that are relevant to gene regulation. For example, A/B compartments are usually identified at megabase scale, whereas topologically associating domains (TADs) are often analyzed using 10- to 50-kb bins (*1*). Significant loop structures can be observed at finer resolutions, using 5- to 10-kb bins. Mapping fine-resolution contact maps can identify enhancer-promoter (E-P) interactions that underlie the cell-type-specific regulation of individual genes, and fine-grained contact maps across developmental stages can reveal the dynamics of enhancer rewiring. However, constructing high-resolution contact maps requires high-complexity and deeply sequenced libraries, which can be costly. Contact maps derived from low-coverage libraries often suffer from high noise due to data sparsity, making it challenging to integrate 3D information into the study of gene regulation.

In recent years, the development of single-cell Hi-C technologies has further enabled the study of 3D genome structures at finer cell-type-specific granularity. This advance in principle enables the analysis of 3D structural heterogeneity and variation in regulatory interactions, even across cells of the same cell type. However, analyzing structures at the single-cell or even pseudobulk level remains difficult due to data sparsity. It is most common to bin single-cell contact maps at very low resolution, such as 100 kb to 1 Mb, or to use of hundreds of thousands of cells to generate pseudobulk data with finer resolutions.

Given the success of deep learning technology for image denoising and image super-resolution (*2*, *3*), several groups have designed supervised deep learning models to denoise bulk Hi-C libraries. HiCPlus (*4*) and HiCNN (*5*) use convolutional neural networks (CNNs) to predict high-coverage contact maps from low-coverage or downsampled contact maps in the same cell type. Meanwhile, hicGAN (*6*), DeepHiC (*7*), and HiCSR (*8*) all use generative adversarial networks (GANs) to impute high-resolution data, with DeepHiC and HiCSR using loss functions specifically tailored to Hi-C data. HiCARN (*9*) uses a cascading neural network to achieve resolution enhancement. Nevertheless, these supervised models often encounter generalization challenges. First, they require paired low/high-coverage Hi-C libraries for training, limiting their applications in scenarios where suitable training data are not available, such as training to predict at ultrahigh resolution where very deeply sequenced libraries are not available or training on single-cell libraries where high-resolution single-cell contact maps are unknown. Second, they may struggle with generalization to new datasets, for example, where the low/high-coverage sequencing depth gap between the input data and desired prediction differs from the training scenario. Furthermore, these approaches usually require specific normalization and preprocessing of the Hi-C datasets, posing additional challenges for the postprediction recovery procedure needed to reconstruct a genome-wide matrix for downstream analysis.

There has been relatively little work on self-supervised denoising strategies, which do not require high-resolution target data during training. Zhang *et al.* (*10*) introduced DeepLoop, a two-step deep learning framework trained in a self-supervised manner to enhance chromatin interaction detection from low-coverage data. DeepLoop begins with smoothed matrices generated by HiCorr (*11*), a Hi-C data bias correction pipeline designed to improve interaction detection. The framework involves training two models: LoopDenoise, an autoencoder that is used to denoise the contact matrix, and LoopEnhance, a U-Net that uses downsampled HiCorr matrices as input examples and is trained in a supervised manner using enhanced matrices produced by LoopDenoise as targets. Although this method improves loop calling from sparse Hi-C matrices, the two-step approach introduces complexity in data preparation and training procedures. The downsampling needed for training LoopEnhance presents a similar challenge to supervised model training, requiring users to train multiple models with different downsampling ratios and to select the one that works best for their data. Moreover, this method has mainly been evaluated for enhancing loop detection but not for denoising other 3D structures like TADs.

For enhancing signals from single-cell Hi-C libraries, Higashi (*12*) and fast-Higashi (*13*) provide fast and efficient algorithms based on hypergraph representation learning, which learns the latent relationship between cells and borrows information from nearby cells to impute cell-type-specific structures. These methods also simultaneously calculate a cell embedding from the imputed 3D structure, providing a convenient option for cell clustering. However, Higashi and fast-Higashi require whole-genome 3D information for imputation, posing a memory challenge, with no lower-cost option to examine cell-to-cell variability at a local region around a gene of interest.

To address these challenges with a single framework, we introduce HiC2Self, a simple and efficient self-supervised Hi-C denoising model. HiC2Self can be widely used for a variety of tasks, including denoising bulk Hi-C data, denoising Micro-C data at ultrahigh resolution, reconstructing cell-cluster-specific contact maps using single-cell Hi-C, and examining local 3D structures at a specific locus or gene at the single-cell level.

## RESULTS

### HiC2Self uses a self-supervised framework to denoise the Hi-C contact maps

**Fig. 1.**
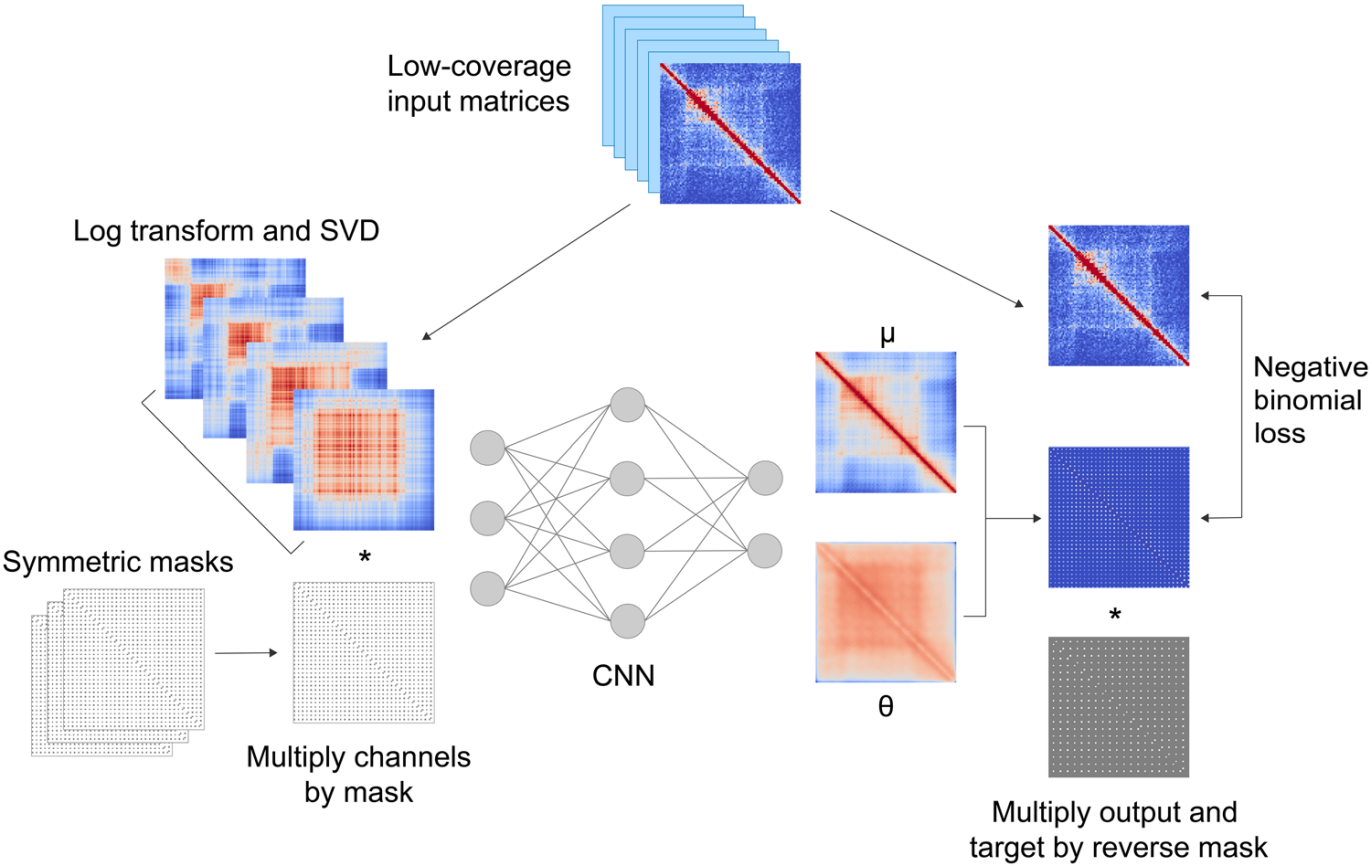
Framework of HiC2Self. HiC2Self uses a self-supervised framework with low-coverage input matrices serving as both the input and the training target, an SVD on low-coverage inputs to enhance low-rank signals, and symmetric masks to force the model to predict masked pixels from unmasked ones.

An overview of the HiC2Self framework is displayed in Fig. 1. There are three key components in the HiC2Self framework:

1) The low-coverage contact matrix is first decomposed and reconstructed using singular value decomposition (SVD) for signal enhancement.

2) A self-supervised training framework adapted from Noise2Self (*14*) is applied to the data, using the low-coverage input matrices as both the input and the target. The model applies diagonally symmetric grid masks to each input channel and learns to predict the masked pixels from the unmasked pixels.

3) The underlying model is a simple CNN that takes in the multichannel tensor as the input and has two output channels, representing the mean and dispersion of the negative binomial (NB) loss, to directly denoise the contact matrices at the level of raw counts.

As shown in Fig. 1, equal-sized low-coverage input matrices are used as both the input and the training target. During training, each of the low-coverage matrices is log transformed for numerical stability and then subjected to SVD to obtain the top eigenvalues and eigenvectors. SVD and low-rank reconstruction is a classic approach for 2D image compression and denoising. We reconstructed low-rank approximations of the matrices using the top *n* eigenvalues and their corresponding eigenvectors (for *n* = 1…4), with each approximation thus capturing increasing levels of variance in the data.

We adapted the Noise2Self (*14*) framework, which uses masks to achieve self-supervised training. Masks are matrices of the same dimension as the low-coverage contact maps, where masked regions corresponding to indices in J have zero entries, and unmasked regions at the complementary indices (those in $J^{C}$) have entries equal to 1. Notationally, we can also identify the set of indices J with the corresponding mask matrix, so that the matrix $J^{C}$ corresponds to the reverse mask (1 − J). Because the contact maps are diagonally symmetric, we designed the masks in a similar manner, ensuring that the entries in the masked and unmasked regions are independent from each other. The mask structure is shown in fig. S1A. Each channel in the multichannel input matrices is multiplied by the mask before going into the model.

HiC2Self uses a simple CNN to denoise the contact matrices and NB loss to train the model. The CNN model consists of five equal-sized convolutional layers, where each of the first three layers is followed by Rectified Linear Unit (ReLU) activation functions, and the last two layers are used in the prediction heads. An exponential function is used at the last layer to transform output values back to the raw count space. We assume the raw counts follow an NB distribution. The model outputs two channels, μ and θ, and we use the negative log-likelihood (NLL) of NB probability as the training objective to optimize the HiC2Self model. During training, both the predicted channels and the low-coverage input matrix need to be multiplied by the reverse (complementary) mask before calculating the loss (Fig. 1).

To evaluate the optimized model structure and assess scalability, we first performed a hyperparameter search on the number of convolutional layers and examined the model performance across varying input matrix sizes. We retrained HiC2Self using one, three (default), and five convolutional layers before the prediction heads, and the results are shown in fig. S1B. HiC2Self with three convolutional layers slightly outperformed the other configurations. We used a two-sided Wilcoxon rank sum test on correlation metrics to assess performance. For Pearson correlation, the *P* values were 3.91 × 10^−15^ for one layer versus three layers, 2.5 × 10^−10^ for three layers versus five layers, and 0.094 for one layer versus five layers. For Spearman correlation, the *P* values were 0.098, 2.3 × 10^−6^ and 0.002 for the same comparisons, respectively.

To further examine the model scalability, we evaluated model performance across different input matrix sizes (200×200, 400×400, 800×800, and 1000×1000), as summarized in table S1. The runtime of HiC2Self increases approximately as O(size^2^), where size represents the number of row/columns, indicating quadratic scaling with respect to input matrix size (and linear scaling with respect to the number of matrix entries). Accordingly, the training resource requirements also grow quadratically with matrix size.

In the default setting, HiC2Self is both trained and evaluated using the masks. To assess the effect of masking ratio on performance and scalability, we retrained HiC2Self using different mask widths: mask width = 3 (one pixel masked out of every three), mask width = 8, and mask width = 12. We found that the model achieved comparable performance across masking ratios, with slightly better results using sparser masks. The results are shown in fig. S1C. A two-sided Wilcoxon rank sum test was used to evaluate differences in Pearson and Spearman correlations: For Pearson correlation, the *P* values were 0.018 for mask 3 versus mask 8, 0.787 for mask 8 versus mask 12, and 0.008 for mask 3 versus mask 12. For Spearman correlation, the corresponding *P* values were 0.924, 0.958, and 0.885, respectively. We further evaluated scalability across different masking ratios, with training and inference times summarized in table S2. We found that sparser masks (i.e., larger mask widths) lead to longer inference times. Considering both performance and computational efficiency, we selected mask width = 3 as the default setting for HiC2Self. In addition, HiC2Self supports one-pass inference without using a mask. We evaluated the prediction accuracy with and without masks and found that HiC2Self produced more accurate predictions when inference was performed with a mask (fig. S1D).

### HiC2Self accurately reconstructs cell-type-specific contact maps from low-coverage libraries

**Fig. 2.**
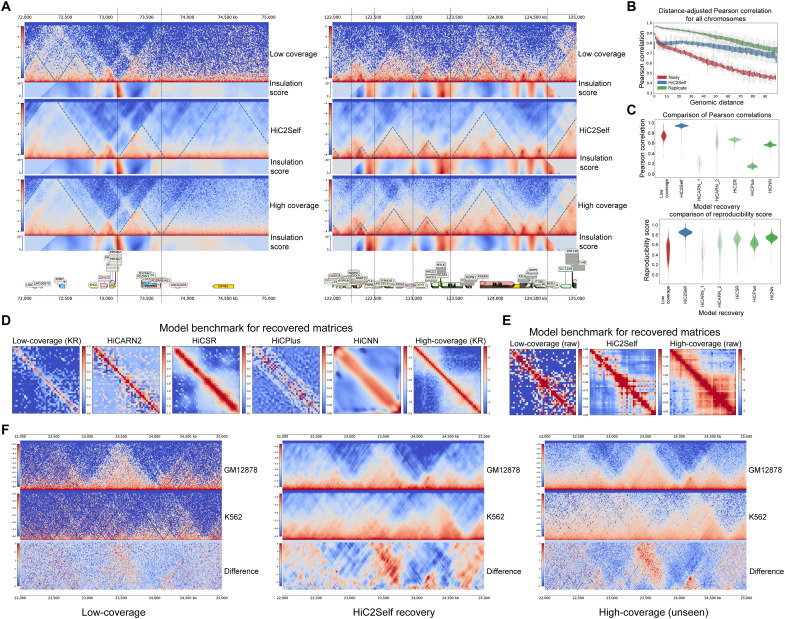
HiC2Self shows robust performance on bulk Hi-C libraries. (**A**) Two random regions on chromosome 3 as a visual comparison example of HiC2Self recovery. The top panel shows the low-coverage Hi-C input, the middle panel shows the HiC2Self recovery, and the bottom shows a deeply sequenced library. The blue dashed triangle lines show the TAD calls identified by TopDom (*16*), and the vertical dashed lines show the TAD boundaries called from the deeply sequenced library. (**B**) Distance-adjusted Pearson correlation of each library compared with the deeply sequenced library, up to 1 Mb from the diagonal. Red boxes show the correlation from the low-coverage library, blue boxes show the HiC2Self recovery, and green boxes show a deeply sequenced biological replicate compared with the high-coverage library. (**C**) HiC2Self benchmark results against supervised models on an unseen new dataset. The top panel shows the Pearson correlation of each predicted library versus the deeply sequenced library. The bottom panel shows the reproducibility score (*18*). Violin plots from left to right: low-coverage contact map, HiC2Self predicted map, supervised models HiCARN [version 1; (*9*)], HiCARN (version 2), HiCSR (*8*), HiCPlus (*4*), and HiCNN (*5*). (**D**) This panel shows an example random region on chromosome 4. From left to right: low-coverage input matrix, recovered matrices from HiCARN version 2, HiCSR, HiCPlus, HiCNN, and the corresponding region in the high-coverage contact matrix. All matrices are KR normalized. (**E**) Same example region with HiC2Self. From left to right: low-coverage contact map, HiC2Self recovery, and high-coverage contact map. All matrices are unnormalized. (**F**) Visual comparison of cell-type-specific recovery of HiC2Self. Low-coverage libraries are on the left, HiC2Self recoveries are in the middle, and high-coverage libraries are on the right. In each panel, from top to bottom: GM12878, K562, and difference between the two libraries.

We first evaluated the denoising performance of HiC2Self on a low-coverage bulk Hi-C library. A relatively shallowly sequenced library on GM12878 [GSE63525; (*15*)] with 202.10 million reads was used as the low-coverage input to HiC2Self, and we compared the results against a pooled high-coverage library with 3.5 billion reads that was never seen by the model. Both libraries were binned at 10-kb resolution, and we performed HiC2Self denoising on the low-coverage library on regions up to 1-Mb distance from the diagonal. Two example regions of HiC2Self recovery on chromosome 3 are shown in Fig. 2A. Within each example, the top row shows the low-coverage contact maps, followed by the insulation score calculated from the overall contact map. The middle row shows the HiC2Self-recovered matrices, and the bottom row shows the unseen pooled high-coverage library. Input and predicted contact maps are Knight-Ruiz (KR) normalized for visualization. From the visual comparison, HiC2Self recovers structures that are consistent with the high-coverage library. We further examined the identification of TADs, called using TopDom (*16*), on the low-coverage, HiC2Self-recovered, and high-coverage contact maps. Blue dashed lines show the TAD calls from each contact map. In these examples, the TAD structure in the HiC2Self-recovered contact maps is consistent with that from the high-coverage library, whereas the low-coverage input data can lead to miscalled TADs.

For a genome-wide evaluation, we then compared the reconstruction of HiC2Self on this low-coverage dataset against the performance of a deeply sequenced biological replicate library with 2.9 billion reads [GSE63525; (*15*)], where again the task was to recover the high-coverage (3.5 billion read) ground truth contact maps. Figure 2B shows a distance-adjusted Pearson correlation across all chromosomes between low-coverage, HiC2Self reconstruction, and biological replicate compared with the high-coverage ground truth library. Red bars show the correlation between original low-coverage and high-coverage maps, green bars show the correlation of the biological replicate with the ground truth, and blue bars show the performance of HiC2Self-recovered maps. Thus, using a library with less than 0.1% reads as input, HiC2Self was able to achieve almost comparable performance to a deeply sequenced biological replicate library. We also normalized the raw and predicted contact maps of chromosome 3 using HiC-DC+ (*17*) and calculated the Receiver Operating Characteristic (ROC) and Precision-Recall (PR) curves of the significant interactions identified by the low-coverage library, HiC2Self, and the biological replicate, compared with the high-coverage unseen library. The results are shown in fig. S2A and confirm that HiC2Self can reconstruct significant interactions from a low-coverage library.

We further benchmarked HiC2Self against previously published supervised models for the bulk library denoising task. We compared HiC2Self with four supervised Hi-C contact map denoising models, including HiCSR (*8*), HiCPlus (*4*), HiCNN (*5*), and HiCARN (*9*). For supervised models, we downloaded pretrained checkpoints of models that were trained on low-coverage and high-coverage contact map pairs based on a 1/64 downsampled sequencing depth ratio. The supervised models were trained on GM12878 Hi-C data with KR normalization at 10-kb resolution, with chromosome 4 held out as the test chromosome. We benchmarked all the models on a new low-coverage GM12878 library (84.91 million reads) representing about 1/42 of the sequencing depth of the deeply sequenced library (3.5 billion reads). Chromosome 4 at 10-kb resolution was used for the evaluation of all models. The supervised models were trained and evaluated using KR normalization, whereas HiC2Self was trained with raw matrices and evaluated on matrices with local KR balancing applied to each matrix. We used Pearson correlation and the GenomeDISCO reproducibility score (*18*) as evaluation metrics. Figure 2C shows the Pearson correlation and reproducibility score comparing low-coverage input library (red), HiC2Self recovery (blue), and pretrained HiCARN, HiCSR, HiCPlus, and HiCNN recovered matrices (green). HiC2Self outperforms all supervised models when generalizing to a new dataset. For a visual comparison of the denoising results, Fig. 2D shows a random example region on the test chromosome (chr4) for all the supervised models (left to right: low-coverage KR normalized matrix, HiCARN2, HiCSR, HiCPlus, HiCNN, and high-coverage KR normalized matrix). Figure 2E shows the same region using HiC2Self recovery (left to right: low-coverage raw matrix, HiC2Self recovery, and high-coverage raw matrix).

We also compared the significant loop calls from HiC2Self with those from a previous self-supervised training framework called DeepLoop. DeepLoop reanalyzed previously published data from Rao *et al.* (*15*) for mid-range cis-interactions (<2 Mb) at 5-kb resolution. For GM12878, five biological replicates were used for training LoopDenoise, with sequencing depth ranging from 252.43 million reads to 1.78 billion reads. The results are available at GSE167200. We retrained HiC2Self using only one replicate with 202.10 million reads [GSE63525; (*15*)] on chromosome 3 at 5-kb resolution, covering 2 Mb from the diagonal, and calculated significant interactions using HiC-DC+ (*17*). The true significant interactions were defined by using HiC-DC+ (*17*) on a deeply sequenced library with 3.5 billion reads. DeepLoop achieved an area under the ROC curve (auROC) of 0.72 and an area under the PR curve (auPR) of 0.34, whereas HiC2Self obtained an auROC of 0.77 and an auPR of 0.40. Therefore, HiC2Self, trained with a single low-coverage replicate achieved slightly better performance than DeepLoop, which was trained with five replicates with deeper sequencing depth (fig. S2B).

As a benefit of its self-supervised training framework, HiC2Self can be easily applied to different datasets and cell types without the generalization challenge often encountered by supervised models. We again ran HiC2Self on the previously used GM12878 libraries as well as the K562 libraries from Rao *et al.* (*15*) and compared the model performance for capturing cell-type-specific structures. The low-coverage K562 library used as input has 79.91 million reads, whereas the high-coverage library has 1.4 billion reads. Figure 2F shows a comparison of a cell-type-specific region on chromosome 3 of GM12878 (top row in each panel), K562 (middle row), and the difference between the two cell types (bottom row). Low-coverage data are shown on the left, with HiC2Self recovery in the middle, and high-coverage data on the right. The distance-adjusted Pearson and Spearman correlations for chromosome 3 in K562 are shown in fig. S2C (left), whereas the distance-adjusted correlations for cell type differences (GM12878-K562) are shown in fig. S2C (right). We also calculated the similarity between GM12878 and K562 using the high-coverage data (green), HiC2Self recovery (blue), and low-coverage data (red), as shown in fig. S2D. This analysis shows that HiC2Self can accurately reconstruct cell-type-specific structures in different cell types.

As a versatile self-supervised model, HiC2Self is not limited to training on a single cell type; it can also be trained on a mixture of cell types while still retaining the ability to recover structures specific to each cell type in the training set. We retrained HiC2Self on a combined dataset containing both GM12878 and K562 samples and compared its performance with models trained on individual cell types. Figure S2E shows this cross-cell-type training comparison. The left panel shows the Pearson correlation of low-coverage GM12878 data (blue), HiC2Self trained on GM12878 (red), HiC2Self trained on K562 (pink), and HiC2Self trained on the GM12878-K562 mixture (pink), all compared against the high-coverage GM12878 reference. The right panel shows the corresponding results for K562. These results show that HiC2Self can accurately reconstruct cell-type-specific structures even when trained on data from a mixture of cell types.

Although HiC2Self is a self-supervised model, we were still interested in evaluating its ability to generalize to new datasets and cell types. We assessed the generalization performance of HiC2Self across GM12878, K562, and a mouse cell line, CH12.LX. The low-coverage CH12.LX library contains 78.65 million reads, whereas the high-coverage library contains 1.4 billion reads. We trained HiC2Self on each individual cell type and cross-validated its performance on the other two cell types. Figure S2F shows the cross-dataset and cross-cell-type generalization results. As expected, HiC2Self achieved the best denoising performance on the cell type it was trained on, with performance declining when applied to other datasets. Notably, generalization performance was highly sensitive to the sparsity of the input data. Therefore, in cross-dataset scenarios, unlike the self-supervised setting for which HiC2Self was designed, the model can be affected by generalization challenges like those encountered by supervised approaches.

### HiC2Self recovers significant interactions at ultrahigh resolution

**Fig. 3.**
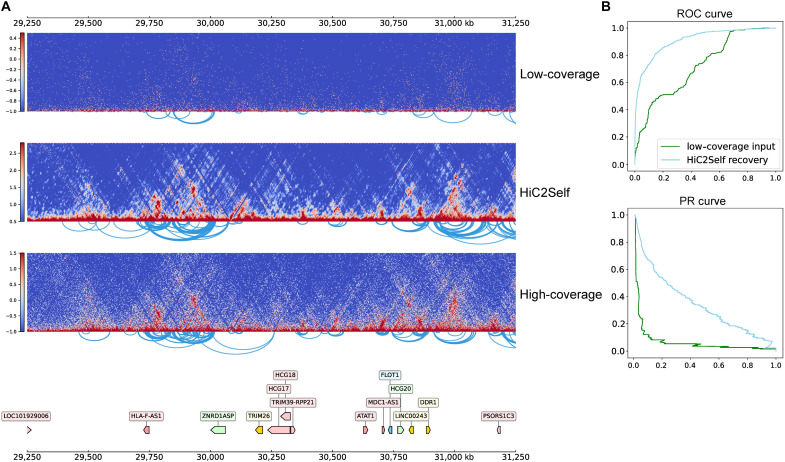
HiC2Self application at ultrahigh resolution. (**A**) Visual comparison of the HLA region (chr6: 29,250,000 to 31,250,000) on chromosome 6 in mESCs. Arcs show the significant interactions identified from each dataset. The significant interactions are defined as top 1% of the distance-adjusted *z*-score. (**B**) ROC and PR curve of significant interactions identified from the low-coverage dataset compared with the deeply sequenced dataset (green) and HiC2Self versus the deeply sequenced dataset (blue).

Given the robust performance at accurate recovery of TADs and loops on bulk Hi-C data, we further explored the ability of HiC2Self to reconstruct 3D structures at finer resolution from Micro-C data. Micro-C (*19*) is an assay to map 3D chromosome conformation that uses MNase instead of restriction enzymes to cut the DNA, enabling it to achieve higher resolution compared to Hi-C libraries. We used a low-coverage H1 embryonic stem cell (hESC) Micro-C library with 188.79 million reads, binned at 1-kb resolution, and ran HiC2Self on chromosome 6 to reconstruct the human leukocyte antigen (HLA) region. A deeply sequenced Micro-C library with 3.4 billion reads was also binned at 1 kb and used as the unseen ground truth to evaluate the reconstruction performance of HiC2Self. Figure 3A shows a visual comparison of the low-coverage library, HiC2Self recovery, and the deeply sequenced library. Notably, HiC2Self successfully reconstructed TAD structures from a very sparse low-coverage library. To quantitatively evaluate the ability of HiC2Self to reconstruct meaningful structures at 1-kb resolution, we calculated a distance-adjusted *z*-score for interaction bins at each genomic distance from the diagonal and defined the top 1% of these *z*-scores as significant interactions in the low-coverage, HiC2Self-recovered, and high-coverage contact maps. The ROC and PR curves of interactions identified from the low-coverage library and HiC2Self versus the high-coverage ground truth are shown in Fig. 3B. Thus, HiC2Self can reconstruct significant interactions from low-coverage Micro-C data at very fine resolution.

We further evaluated HiC2Self’s ability to reconstruct signals from Micro-C data by comparing its output to Region Capture Micro-C (RCMC) data (*20*). We used a standard mouse embryonic stem cell (mESC) Micro-C library with 1.3 billion reads, binned at 500–base pair (bp) resolution, and ran HiC2Self on chromosome 3 to cover the *Sox2* region. Figure S3A shows a comparison of the raw Micro-C data, HiC2Self reconstruction, and RCMC data at a 400-kb region around the *Sox2* locus. HiC2Self slightly improved matrix coverage in sparse regions. However, it was unable to impute signals that were entirely absent from the input matrix and uniquely captured by RCMC.

In addition, we evaluated whether HiC2Self can reconstruct meaningful structures from Hi-C data at ultrahigh resolution. We processed a deeply sequenced K562 Hi-C library at 500-bp resolution and compared the HiC2Self-reconstructed contact maps with CRISPR-FlowFISH results (*21*) for E-P interactions. Figure S3B shows the HiC2Self reconstruction results for E-P regions of the *HBG1* (left) and *FEZ1* (right) genes. In each panel, the top row shows the sparse input matrix, where individual dots represent single reads due to extreme sparsity. The middle row shows the HiC2Self-reconstructed contact maps, and the bottom row shows CRISPR-FlowFISH–validated E-P interactions as arcs. Despite the extremely sparse input, HiC2Self is able to recover major chromatin structures at 500-bp resolution on Hi-C contact maps. Meanwhile, as we have seen in the previous examples, regions that are unmappable or have no signals in the input matrices cannot be imputed by the model.

### HiC2Self accurately reconstructs cell-population-specific 3D genome structures with single-cell Hi-C

In addition to achieving robust performance for denoising bulk Hi-C libraries, HiC2Self can also enhance 3D structures from single-cell Hi-C libraries, facilitating the examination of contact maps at the pseudobulk or single-cell level. Single-cell Hi-C technology has progressed rapidly in recent years. In particular, the emergence of various single-cell multimodal co-assays allows the definition of cell clusters or cell types based on the better understood expression or epigenomic readouts, followed by the examination of cell-cluster-specific pseudobulk 3D structures at high granularity. Single-nucleus methyl-3C sequencing (sn-m3C-seq) (*22*) is a single-cell multimodal assay that simultaneously measures chromosome conformation and DNA methylation and was recently used to profile 53,000 cells from the human prefrontal cortex (PFC) and hippocampus (HPC) to investigate the epigenome and 3D genome during brain development. In this study, cell types were determined on the basis of clustering and annotation of their methylation profiles.

**Fig. 4.**
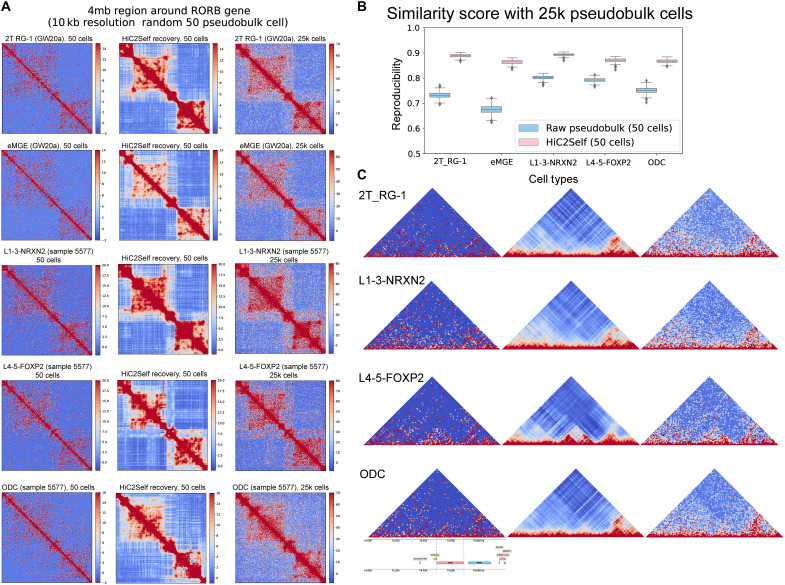
HiC2Self recovers cell-population-specific 3D structures from single-cell Hi-C libraries. (**A**) Comparison of a 4-Mb region around the *RORB* gene, with pseudobulk libraries binned at 10-kb resolution. The left column shows the pseudobulk map based on 50 cells, the middle column shows the HiC2Self recovery, and the right column shows the higher coverage pseudobulk map based on 25,000 cells. Different cell types are shown from top to bottom: 2T_RG-1 (radial glia cells in the second trimester), eMGE_GW20a (MGE during gestational week 20), L1-3-NRXN2, L4-5-FOXP2, and ODC_5577 (ODCs). (**B**) Similarity score comparing 50-cell pseudobulk maps versus the 25,000-cell pseudobulk maps (blue) and the HiC2Self-recovered 50-cell maps versus the 25,000-cell pseudobulk maps (pink). (**C**) Zoomed-in visualization of a 1-Mb region around the *RORB* gene, where each row shows the cell-type-specific 50-cell pseudobulk, HiC2Self recovery, and 25,000-cell pseudobulk structures. The gene annotation track is shown at the bottom.

Using this dataset, we first evaluated the ability of HiC2Self to reconstruct cell-cluster-specific structures around genes of interest based on sparse pseudobulk input data over a few dozen cells. We examined a 4-Mb local region around *RORB*, an important gene related to schizophrenia, during early human brain development. We randomly sampled 50 cells from each cell subtype in the PFC, including radial glia cells in the second trimester (2T-RG-1) and medial ganglionic eminence (eMGE) at the prenatal stage, and layer 1–3 cortical neurons expressing *NRXN2* (L1-3-NRXN2), layer 4–5 neurons expressing *FOXP2* (L4-5-FOXP2), oligodendrocyte cells (ODCs) at the adult stage, and binned the 50-cell pseudobulk contact maps at 10-kb resolution. We then trained HiC2Self on chromosome 9 of this 50-cell pseudobulk input contact map. Figure 4A shows a visual comparison of the 4-Mb region around the *RORB* gene, with the raw 50-cell pseudobulk maps on the left, HiC2Self-recovered maps in the middle, and 25,000-cell pseudobulk maps for each cell type as the ground truth on the right. A similarity score comparison of the raw 50-cell pseudobulk map and HiC2Self recovery versus the 25,000-cell pseudobulk map is shown in Fig. 4B. Furthermore, zooming into a 1-Mb region around the *RORB* gene, it is clear that HiC2Self successfully reconstructed the distinct structure at the L4-5-FOXP2 cell stage with as little as 50 cells. Thus, HiC2Self applied to pseudobulk maps of a few dozen cells enables the examination of 3D genome folding structures and regulatory interactions around genes of interest in rare cell types from single-cell Hi-C datasets.

HiC2Self can robustly reconstruct cell-type-specific structures in single-cell Hi-C data at the pseudobulk level, and we systematically evaluated the model’s robustness through several analyses. First, we assessed reproducibility in 50-cell pseudobulk sampling. In the earlier example, HiC2Self was trained on a mixture of multiple cell types, each consisting of 400 samples of 50 pseudobulk cells. For this reproducibility test, we selected a single cell type and generated three independent replicates by resampling 400 pseudobulk samples, retraining the model each time, with results shown in fig. S4A. The left panel shows the Pearson correlations between raw pseudobulk maps (blue) and HiC2Self reconstructions (pink) compared to the ground truth. The right panel shows the distribution of Pearson correlations across HiC2Self reconstructions. HiC2Self showed consistent performance across multicell-type versus single-cell-type training, as well as across different resampled subsets of cells.

We further evaluated the impact of read depth on model performance while keeping the number of cells constant (fig. S4B). The top panel shows the correlation between read depth and agreement with the 25K-cell pseudobulk ground truth in raw 50-cell pseudobulk maps, where higher read counts lead to improved correlation. The bottom panel shows the performance of HiC2Self reconstructions, where all samples achieved correlations ranging from 0.85 to 0.95, indicating that HiC2Self reduces variability due to sequencing depth and enhances consistency across samples. Last, we evaluated the effect of pseudobulk cell count on reconstruction performance. Figure S4C shows the results for HiC2Self trained on pseudobulk samples containing 10, 25, 50, or 100 cells, all from the 2T-RG-1 cell type and binned at 10-kb resolution. As expected, increasing the number of cells (and thereby read depth) in the raw input improves correlation with the ground truth, whereas HiC2Self consistently achieved robust performance across all tested cell counts, demonstrating its reliability even with low-cell input data.

In addition, we evaluated whether using the loss function of an alternative count distribution could further improve HiC2Self performance under highly sparse conditions. Although HiC2Self models contact counts using an NB loss to account for overdispersion, single-cell Hi-C and low-cell-count pseudobulk data often show a high degree of sparsity. To assess the potential benefit of explicitly modeling zero inflation, we retrained HiC2Self using a zero-inflated negative binomial (ZINB) loss and compared it to the default NB results. We again used the pseudobulk samples derived from the 2T-RG-1 cell type with 100, 50, and 10 cells, where sparsity is progressively more pronounced. As shown in fig. S4D, correlation-based reproducibility scores were consistently higher for HiC2Self reconstructions rather than for the corresponding raw pseudobulk inputs across all cell counts. Specifically, for the 100-cell and 50-cell pseudobulk settings, the NB-based HiC2Self model outperformed ZINB-based models, whereas in the extremely sparse 10-cell setting, the two loss functions performed comparably.

A visual comparison of reconstructed contact maps further supports the quantitative evaluations (fig. S4E). For all pseudobulk sizes, NB-based and ZINB-based HiC2Self reconstructions were visually similar, and both substantially enhanced contact map structures relative to the raw inputs. Together, these results indicate that, although zero-inflated formulations can perform comparably under extreme sparsity, the NB loss provides robust performance across a broad range of pseudobulk settings, supporting its use as the default loss function in HiC2Self.

### HiC2Self reconstructs single-cell 3D structures without borrowing information from neighboring cells

**Fig. 5.**
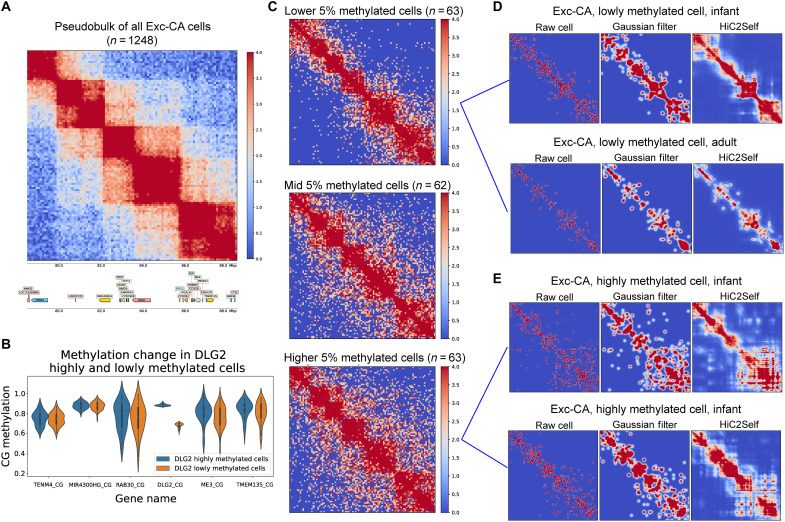
HiC2Self reconstructs the single-cell 3D structures. (**A**) Region (20 Mb) around the *DLG2* gene from a pseudobulk contact map of 1248 Exc-CA cells binned at 50-kb resolution. The gene annotation track is shown at the bottom. Mbp, mega–base pairs. (**B**) mCG methylation level of *DLG2* and nearby genes (including *TENM4*, *MIR4300HG*, *RAB30*, *PDLG2*, *ME3*, and *TMEM135*) from the lowest 10% *DLG2*-methylated cells (orange) versus the highest 10% *DLG2*-methylated cells (blue). (**C**) Comparison of the 20-Mb region around the *DLG2* gene from the pseudobulk contact map with the lower 5% *DLG2*-methylated cells (top), middle 5% *DLG2*-methylated cells (middle), and highest 5% *DLG2*-methylated cells (bottom). Each pseudobulk library consists of 62 to 63 cells. (**D**) Two randomly sampled cells from the lowest 5% *DLG2*-methylated cells, with the raw cell contact matrix on the left, Gaussian-smoothed matrix in the middle, and HiC2Self-recovered matrix on the right. (**E**) Two randomly sampled cells from the highest 5% *DLG2*-methylated cells, with the raw cell matrix on the left, Gaussian-smoothed matrix in the middle, and HiC2Self recovery on the right.

Given the strong performance of HiC2Self in recovering structures from low-cell-number pseudobulk maps in single-cell Hi-C datasets, we tested the application of HiC2Self at the single-cell level. In the human cortex sn-m3C-seq dataset (*22*), we noticed that some genes (e.g., *DLG2* gene) show strong variability in methylation levels even within the same cell type. Therefore, in addition to reconstructing the cell-type-specific 3D structures, we investigated whether HiC2Self could capture cell-to-cell variation in the local 3D structure around genes of interest in individual cells of the same cell type. Figure 5A shows a 20-Mb region around the *DLG2* gene using a pseudobulk map of 1248 Exc-CA (excitatory neurons from the cornu ammonis regions) cells from the HPC region, binned at 50-kb resolution. Figure 5B shows the methylation level of *DLG2* and nearby genes from the top 5% highest *DLG2*-methylated cells versus the bottom 5% *DLG2*-methylated cells. We can see that, even within the same cell type, cells present diverse methylation patterns for certain genes.

Figure 5C shows the 3D structure of a 20-Mb region around the *DLG2* gene from the 5% lowest methylated cells (top), the middle 5% of cells with intermediate methylation (47.5 to 52.5%; middle), and 5% highest methylated cells (bottom). Cells with higher methylation at *DLG2* tend to form longer-range interactions compared with cells with low methylation at this gene. Figure 5D shows two random cells sampled from the 5% lowest *DLG2*-methylated cells, with the sparse raw single-cell contact map on the left, Gaussian-smoothed map in the middle, and HiC2Self-reconstructed single-cell map on the right. Figure 5E shows two random cells sampled from the 5% highest *DLG2*-methylated cells. We further calculated a Uniform Manifold Approximation and Projection (UMAP) using the raw single-cell (top) and the HiC2Self-reconstructed (bottom) local contact maps, shown left to right in fig. S5A: the UMAP colored by developmental stage, contact scores reported from Heffel *et al.* (*22*), and mCG and mCH methylation of the *DLG2* gene. The UMAP shows that cells grouped by the local 3D structure, as recovered by HiC2Self around the *DLG2* gene, show a pattern consistent with its methylation levels. We further calculated the pairwise cosine similarity of the normalized cells across all cell types and compared with the pairwise distance of CG methylation around the *DLG2* genes, as shown in fig. S5B. The reproducibility score (*18*) of the cosine similarity matrix is 0.83 between the raw cell matrix and the CG methylation matrix and 0.95 between the HiC2Self recovery and the mCG matrix. HiC2Self-reconstructed cells thus show a more consistent correlation between pairwise contact map similarity with CG methylation. HiC2Self thus revealed cell-to-cell variability in 3D structure within the same cell type that correlated with the methylation pattern, making possible the study of single-cell gene regulation patterns among cells of the same cell type.

In addition to capturing cell-to-cell variation in local 3D structures around genes of interest, we investigated whether HiC2Self could enhance the identification of higher-order chromatin organization, specifically A/B compartments, from single-cell Hi-C data. To this end, we analyzed single-cell Hi-C datasets from human hippocampal progenitor cells, comprising five samples (GW18-Ant, GW18-Post, UMB4267, UA19, and UMB6096). For each single cell, chromosome 3 was binned at 100-kb resolution, yielding contact matrices of size 1983 × 1983. We retrained HiC2Self on a mixed dataset of over 5000 single cells from all samples and generated denoised contact maps for each individual cell. Compartment (PC1) scores were then computed on a per-cell basis from the raw single-cell matrices, the HiC2Self-recovered matrices, and the corresponding pseudobulk reference constructed from all cells of each sample.

We randomly sampled 5% of cells from each sample and compared the compartment scores of individual cells with the corresponding pseudobulk references using both Pearson and Spearman correlation. Figure S5C shows the distribution of correlations across all sampled cells, where HiC2Self-recovered single-cell contact maps showed significantly higher agreement with the pseudobulk compartment profiles than raw single cells (Wilcoxon rank sum test *P* = 6.28 × 10^−61^ for Pearson correlations and *P* = 2.29 × 10^−31^ for Spearman correlations). Across all samples, HiC2Self improved the maximum Pearson correlation ranging between 0.09 and 0.38 in raw single cells to a range of 0.41 to 0.69 after denoising while also increasing the minimum correlations, indicating more stable compartment inference across cells. Figure S5D highlights two representative single-cell examples, showing the cells with highest and lowest Pearson correlations between the compartment scores calculated from HiC2Self-recovered single-cell maps and the pseudobulk compartment scores. Together, these results show that HiC2Self enables reliable inference of A/B compartment organization at the single-cell level without borrowing information from neighboring cells, extending its utility beyond local structural features to higher-order chromatin domains.

Last, we benchmarked HiC2Self with a previously published model for single-cell Hi-C imputation called Higashi (*12*), evaluating on chromosome 1 of the sn-m3C-seq PFC dataset (*22*) at 500-kb resolution. We trained HiC2Self with only one chromosome, whereas Higashi incorporates all the chromosomes during training. Figure S6A shows two random example cells from the Exc_L1-3 (excitatory neurons localized in cortical layers 1–3) cell type at 500-kb resolution, with (left to right) as raw single-cell contact map, HiC2Self recovery, Higashi recovery, and pseudobulk of all the cells from this cell type. We further used distance-adjusted correlation and the reproducibility score to evaluate the performance of two models, with results shown in fig. S6C. In this comparison, we selected three cell types with distinct 3D structures, namely, Exc_L1-3, Inh_MGE (inhibitory neurons originating from the MGE), and NonN_Astro (nonneuronal astrocytes), and evaluated all the cells from each of these three cell types against the pseudobulk of all three cell types to see whether the models could reconstruct cell-type-specific structures and whether reconstructed single-cell maps were most similar to the pseudobulk map of the corresponding cell type.

The top row (fig. S6C) shows the averaged distance-adjusted Spearman correlation, which is calculated for each single cell by first calculating the correlation at each genomic distance from the diagonal and then averaging them to obtain an overall distance-adjusted Spearman correlation for that cell. The bottom row shows the reproducibility score. From left to right are the performance of raw single-cell contact maps, Gaussian-smoothed maps, HiC2Self recovery, and Higashi recovery. In each panel, boxes show the correlation or similarity of all the cells from Exc_L1-3, Inh_MGE, NonN_Astro versus pseudobulk of Exc_L1-3, and all the cells from Exc_L1-3, Inh_MGE, NonN_Astro versus pseudobulk of Inh_MGE, etc. Using distance-adjusted Spearman correlation, all the cell types showed strong cell-type-specific structures, where HiC2Self-recovered single-cell maps had the strongest correlation with the pseudobulk of the corresponding cell type. Higashi had the highest Spearman correlation compared with other methods. Using the reproducibility score, however, HiC2Self slightly outperformed Higashi in similarity to the correct pseudobulk cell type (*P* = 3.55 × 10^−161^, Wilcoxon one-sided rank sum test), as well as giving superior cell type specificity (i.e., higher similarity to the corresponding pseudobulk map than those of other cell types).

## DISCUSSION

In this study, we present HiC2Self, a simple and efficient self-supervised method designed to denoise bulk and single-cell Hi-C contact maps to empower downstream analyses. The HiC2Self framework includes three key components: (i) a CNN with SVD-reconstructed channels for structural enhancement of input contact maps, (ii) a self-supervised framework involving masks to use low-coverage data as both the input and the training target, and (iii) an NB loss to run the model with raw count matrices. In the Results section, we have shown that HiC2Self can accomplish various tasks, including denoising low-coverage bulk Hi-C data, recovering significant interactions at ultrahigh resolution from Micro-C data, reconstructing cell-population-specific 3D genome structures in low-cell-number pseudobulk maps from single-cell Hi-C, and examining gene regulatory patterns at genes of interest at the single-cell level.

In addition to using the NLL of NB probability as the training loss as discussed earlier, we also experimented with different loss functions. We first replaced the NB loss with the structural similarity index measure (SSIM) (*23*) loss instead. SSIM is a perceptual metric to measure the similarity between two images, which is commonly used in the computer vision domain. This loss prioritizes the maintenance of structural similarity instead of penalizing equally for pixel-wise differences. In particular, we applied the SSIM loss on our single-cell datasets and found a notable capacity to recover intricate global structures, as compared with primarily local pattern recovery using traditional NLL loss. However, although the enhanced structures looked promising, they appeared exaggerated in regions with high sparsity, where, potentially, there was insufficient information to correctly recover the true structures. We also applied SSIM loss to bulk Hi-C contact maps and compared with the unseen deeply sequenced ground truth maps and found that the performance was not as good as the NLL loss, potentially indicating the need for more hyperparameter tuning. Therefore, we retained the NLL of NB loss function for all denoising tasks.

Inspired by the SSIM loss design, we further experimented with training the model in a multiresolution manner to capture both local and global structures. In this setting, we not only optimized the loss function on the original resolution to capture local structures but also performed max-pooling on the prediction and target matrices and then optimized the loss on the lower dimensional matrices to capture global structures. We applied this design to both bulk and single-cell applications and found that its performance varied across datasets but was overall comparable to single-resolution training. As a result, we opted to use single-resolution training as the default while providing multiresolution training as an optional feature in the codebase.

Although HiC2Self is capable of addressing numerous applications, it also displays certain limitations. First, because of the design of the SVD-reconstructed channel and masks, HiC2Self requires diagonally symmetric contact maps as input. This design may increase the computational burden when we want to recover long-distance structures as a bigger input matrix is required to train the model. This design also brings difficulties with denoising trans-contacts, where symmetric matrices are not available, although an analogous self-supervised strategy may be feasible. Second, although HiC2Self shows strong performance for reconstructing structures from sparse contact maps, it cannot impute structures that are missing due to limitations of the sequencing assay. For example, when reconstructing 3D structures at 1-kb resolution, HiC2Self cannot impute the unmappable regions from Hi-C libraries. Further model development to address these challenges would be of interest.

In recent years, transformer architectures have been widely adopted in the computer vision field, with models such as Vision Transformer (ViT) (*24*) achieving significant improvements in capturing detailed structures. Building on this progress, the HiCFoundation model (*25*) has introduced transformer-based architectures into the analysis of Hi-C data. A potential future direction for HiC2Self could involve incorporating more advanced architectures such as ViT. Attention-based models may enhance performance over convolutional networks, particularly in capturing long-range and fine-grained structures in sparse Hi-C contact maps. Although stacking deeper convolutional layers in HiC2Self increases the receptive field, convolutional networks are inherently local in nature. In contrast, transformers can directly model long-range dependencies, potentially improving recovery of distal chromatin interactions. However, adopting transformer architectures comes with challenges. As shown in our scalability analyses, training ViT models may require substantially more computational resources and larger datasets. Moreover, ViT is often more susceptible to overfitting, especially when trained across multiple cell types. The patching scheme required by ViT (e.g., 16×16 tiles) may not always align well with the underlying biological signals in contact maps, necessitating custom architectural adjustments for Hi-C data. In summary, although incorporating a ViT architecture could enhance HiC2Self’s ability to capture long-range and high-resolution chromatin features, it would require careful and extensive model design and tuning.

In conclusion, we have presented HiC2Self, an efficient self-supervised deep learning framework designed to denoise Hi-C contact maps. HiC2Self’s denoising strategy includes three key design decisions: a convolutional model with SVD-reconstructed channels, a self-supervised training framework achieved by masks, and NB training loss to train on raw count matrices. HiC2Self can be widely used in a variety of tasks, including denoising bulk Hi-C datasets, reconstructing biologically meaningful 3D structures from Micro-C data at high resolution, as well as reconstructing pseudobulk or single-cell 3D structures to study cell-to-cell variability in single-cell Hi-C datasets. HiC2Self thus provides a flexible denoising tool to enhance the interpretation of single-cell and bulk 3D genomics datasets.

## MATERIALS AND METHODS

### Ethics statement

This study used only publicly available datasets and did not involve any new animal experiments. All data used in this study were obtained from publicly available sources (e.g., ENCODE and GEO) and were generated in accordance with the relevant institutional and national ethical guidelines as described in the original publications.

### Data availability

This study did not generate new materials. All data analyzed in this study were obtained from publicly available sources and are accessible as detailed below.

#### Bulk GM12878 dataset

High-coverage Hi-C datasets are generated by sequencing multiple libraries and aggregating read counts across libraries. To obtain low-coverage Hi-C training data, we generated a contact map from a single library of a high-coverage Hi-C dataset and evaluated performance against the aggregated multilibrary map. Intrachromosomal Hi-C raw count contact maps were generated without normalization. For each chromosome in the low-coverage dataset, we further extracted equal-sized square submatrices along the diagonal, representing genomic interactions up to 1 Mb in linear distance. These symmetric submatrices X were used as the training set for our model. HiC2Self was trained and evaluated on low-coverage and high-coverage Hi-C data, respectively, as described above. Low/high-coverage raw count matrices for the ENCODE GM12878 cell line were downloaded from the GEO [GSE63525, https://ncbi.nlm.nih.gov/geo/query/acc.cgi?acc=GSE63525; (*15*)]. A single low-coverage library (experiment HIC001, https://ncbi.nlm.nih.gov/geo/query/acc.cgi?acc=GSM1551550) with 202.10 million reads was used as low-coverage data to train the model, and pooled primary libraries with 3.5 billion reads (low-to-high ratio = 1/18) were used as high-coverage Hi-C data to evaluate model performance. Raw count data were downloaded in .hic format and further binned at 10-kb resolution matrix using Juicer (*26*). Libraries were aligned to hg19. Equal-sized (200 × 200) submatrices were extracted along the diagonal from intrachromosomal low-coverage Hi-C contact maps to train the model.

#### Bulk K562 dataset

Low/high-coverage raw count matrices for the K562 cell line were downloaded from the GEO [GSE63525, https://ncbi.nlm.nih.gov/geo/query/acc.cgi?acc=GSE63525; (*15*)]. The low-coverage library (experiment HIC071, https://ncbi.nlm.nih.gov/geo/query/acc.cgi?acc=GSM1551620) with 79.9 million reads was used as low-coverage input for model training, whereas the pooled high-coverage library with 1.4 billion reads (low-to-high ratio = 1/17) was used to evaluate model performance. Both libraries were aligned to hg19, and the matrices were processed in the same way as the bulk GM12878 data described above.

#### Bulk CH12.LX dataset

Low/high-coverage raw count matrices for the CH12.LX cell line were downloaded from the GEO [GSE63525, https://ncbi.nlm.nih.gov/geo/query/acc.cgi?acc=GSE63525; (*15*)]. The low-coverage library (experiment HIC091, https://ncbi.nlm.nih.gov/geo/query/acc.cgi?acc=GSM1551636) with 78.65 million reads was used as low-coverage input for model training, whereas the pooled high-coverage library with 1.4 billion reads (low-to-high ratio = 1/18) was used to evaluate model performance. Both libraries were aligned to mm9, and the matrices were processed in the same way as the bulk GM12878 data described above.

#### DeepLoop analysis results on GM12878

DeepLoop (*10*) reanalysis of previously published Hi-C libraries [GSE63525, https://ncbi.nlm.nih.gov/geo/query/acc.cgi?acc=GSE63525; (*15*)] can be found on GSE167200 (https://ncbi.nlm.nih.gov/geo/query/acc.cgi?acc=GSE167200). We downloaded the file GSE167200_GM12878.in_situ.raw_HiCorr_LoopDenoise.txt.gz for the reported loop calls on the GM12878 in situ library.

#### Bulk Micro-C library for ultrahigh-resolution denoising

Both the low-coverage and high-coverage libraries are downloaded from the 4DN data portal (*27*). A high-coverage library on H1 derived differentiated endoderm was downloaded from 4DNESW1SPPTD (https://data.4dnucleome.org/experiment-set-replicates/4DNESW1SPPTD/) and contained 3.4 billion reads, whereas a low-coverage Micro-C library was downloaded from 4DNESP4MARXG (https://data.4dnucleome.org/experiment-set-replicates/4DNESP4MARXG/) and contained 188.79 million reads. These libraries were aligned to hg38. The contact maps were unnormalized and binned at 1-kb resolution, where equal-sized square matrices along the diagonal were extracted from chromosome 6. Each matrix had a shape of 400 × 400, which covered 400 kb from the diagonal. In the evaluation of significant interactions, we calculated a distance-adjusted *z*-score [(observed counts − average counts for that distance)/(SD of counts for that distance)] and defined significant interactions as the top 1% of the interactions from each library.

#### Mouse Micro-C and RCMC data for ultrahigh-resolution denoising

Mouse mESC Micro-C library with 1.3 billion reads was downloaded from the GEO [GSE130275, https://ncbi.nlm.nih.gov/geo/query/acc.cgi?acc=GSE130275; (*19*)]. The library was aligned to mm10. The contact maps were unnormalized and binned at 500-bp resolution, and each matrix has a shape of 800 × 800, covering 400 kb from the diagonal. The RCMC data was downloaded from the GEO [GSE207225, https://ncbi.nlm.nih.gov/geo/query/acc.cgi?acc=GSE207225; (*20*)].

#### Deeply sequenced K562 Hi-C library and the CRISPR-FlowFISH experiments

The deeply sequenced K562 in situ Hi-C library for ultrahigh-resolution reconstruction was downloaded from the 4DN data portal [4DNESI7DEJTM, https://data.4dnucleome.org/experiment-set-replicates/4DNESI7DEJTM/; (*15*)]. Hi-C contact maps were binned at 500-bp resolution. CRISPR-FlowFISH data in K562 cells was downloaded from EPCrisprBenchmark_ensemble_data_GRCh38.tsv.gz [https://github.com/EngreitzLab/CRISPR_comparison/tree/main; (*21*)].

#### sn-m3C-seq dataset

An sn-m3C-seq dataset of human brain development was downloaded from GSE213950 (https://ncbi.nlm.nih.gov/geo/query/acc.cgi?acc=GSE213950) with hg38 alignment. The human sample and metadata for cell types were matched from table S1 and S2 from the original publication by Heffel *et al.* (*22*). For the HPC data, we downloaded specimen UA19-27 (https://ncbi.nlm.nih.gov/geo/query/acc.cgi?acc=GSM6596812), UA18-22 (https://ncbi.nlm.nih.gov/geo/query/acc.cgi?acc=GSM6596827), GW18-Ant (https://ncbi.nlm.nih.gov/geo/query/acc.cgi?acc=GSM6596823), GW18-Post (https://ncbi.nlm.nih.gov/geo/query/acc.cgi?acc=GSM6596824), GW20 (https://ncbi.nlm.nih.gov/geo/query/acc.cgi?acc=GSM6596811), and UMB4267 (https://ncbi.nlm.nih.gov/geo/query/acc.cgi?acc=GSM6596809). We randomly sampled 50 cells from each cell type and extracted equal-sized (400 × 400) matrices around the *RORB* gene on chromosome 9 as training data. For the PFC data, we downloaded specimen UMB1863 (https://ncbi.nlm.nih.gov/geo/query/acc.cgi?acc=GSM6596794), UMB5577 (https://ncbi.nlm.nih.gov/geo/query/acc.cgi?acc=GSM6596799), and GW20a (https://ncbi.nlm.nih.gov/geo/query/acc.cgi?acc=GSM6596796), and only Exc-CA cells were extracted for the study.

#### sn-m3C-seq PFC dataset

The sn-m3C-seq PFC dataset (*22*) used to benchmark with Higashi was downloaded from the preprocessed data in the Higashi tutorial (https://github.com/ma-compbio/Higashi/tree/main/tutorials). We converted the pairs file into contact matrices and extracted equal-sized (400 × 400) matrices along the diagonal to train HiC2Self.

### Model availability

Data preparation pipeline and model scripts are available at Zenodo https://zenodo.org/records/18264342 [DOI: 10.5281/zenodo.18264342; (*29*)] and https://github.com/LeslieLabMSK/HiC2Self.

### Self-supervised framework

Noise2Self (*14*) is a self-supervised denoising framework that uses J-invariant functions f, where J represents the partition of the input data dimensions *m* into subsets, and we consider a subset JϵJ and its complement $J^{C}$. Given an unseen clean signal, $y\epsilon {\mathbb{R}}^{m}$, we assume that x is a mean-zero noisy observation, where E[x∣y]=y. For any fixed subset J, we further assume that a noisy observation on subspace $x_{J}$ is independent of the one on its complement $x_{J^{C}}$ given y. With these two assumptions, a function $f:{\mathbb{R}}^{m}\to {\mathbb{R}}^{m}$ is defined as J-invariant if $f{\left(x\right)}_{J}$ is independent of $x_{J}$ for every JεJ.

The ordinary denoising loss function is defined as

$$\begin{array}{c} L_{f}=E_{x,y}\|f\left(x\right)-{y\|}^{2}\\ =E_{x}\|f\left(x\right)-{x\|}^{2}+{\left\|x-y\right\|}^{2}-2\langle f\left(x\right)-x,x-y\rangle \end{array}$$ (1)

which is the sum of a self-supervised loss and the variance of the noise. With a J-invariant function f and the previous assumptions, this simplifies to

$$L\left(f\right)=\sum_{J\epsilon J}E\|f_{J}\left(x_{J^{C}}\right)-{x_{J}\|}^{2}$$ (2)

so that the denoising function f can be optimized using only noisy observations x.

The J-invariance property is realized using masks. We denote the masked area as $x_{J}$ and the unmasked area as $x_{J^{C}}$. Given the symmetric nature of Hi-C contact maps and the requirement that $x_{J}⫫x_{J^{C}}|y$, we designed masks that are symmetric with respect to the diagonal.

### Model architecture

HiC2Self uses a simple CNN, as shown in Fig. 1. Within the model, raw count input matrices *X* were first log_2_ transformed [$X^{\prime }={\text{log}}_{2}\left(X_{J^{C}}+1\right)$] to guarantee numerical stability for subsequent steps.

SVD and low-rank reconstruction is a classic approach for 2D image compression and denoising. To enhance the low-rank structures extracted from low-coverage submatrices in the log_2_-transformed space, we performed SVD on the log_2_-transformed matrices $X^{\prime }=U\sum U^{T}$, generated reconstructions $X_{k}^{\prime }=\sum_{i=1}^{k}u_{i}\sum_{i}u_{i}^{T}$ using the top k eigenvectors, kϵ[1,4], and concatenated these matrices with *X′* as additional input channels for the CNN.

The convolutional part of the model consists of five equal-sized convolutional layers, where each of the first three layers is followed by ReLU activation functions. An exponential function was used as the activation function for layers 4 and 5 to transform output values back into raw count space.

### Loss function

#### NLL of NB loss

Inspired by the deep count autoencoder (DCA) model for single-cell data (*28*), we used an NB loss for the raw count matrices to train our model. We assume that the count from each bin ($x_{\mathrm{ij}}$) of the contact map *X* follows an NB distribution with parameters $\mu_{\mathrm{ij}}$ and $\theta_{\mathrm{ij}}$, $x_{\mathrm{ij}}∼\text{NB}\left(\mu_{\mathrm{ij}},\theta_{\mathrm{ij}}\right)$. The loss function is defined as

$$\begin{array}{c} L\left(f\right)=-\text{log}L_{\text{NB}}\\ =\sum_{\mathrm{ij}}\left[\text{logΓ}\left(x_{\mathrm{ij}}+1\right)+\text{logΓ}\left(\theta_{\mathrm{ij}}\right)-\text{logΓ}\left(x_{\mathrm{ij}}+\theta_{\mathrm{ij}}\right)+\theta_{\mathrm{ij}}\text{log}\left(\frac{\mu_{\mathrm{ij}}+\theta_{\mathrm{ij}}}{\theta_{\mathrm{ij}}}\right)+x_{\mathrm{ij}}\text{log}\left(\frac{\mu_{\mathrm{ij}}+\theta_{\mathrm{ij}}}{\mu_{\mathrm{ij}}}\right)\right] \end{array}$$ (3)

As shown in Fig. 1, HiC2Self outputs two channels, corresponding to μ and θ in the loss function above. We use $\mu_{\mathrm{ij}}$, the expected value for each bin $x_{\mathrm{ij}}$, as the predicted value for our denoising results.

### Genome-wide prediction

HiC2Self produces denoised results as raw counts for fixed-sized submatrices, which can easily be assembled into a whole-chromosome prediction. To do this, we extracted submatrices along the diagonal, consecutively striding by one bin each time. Denoised results were generated for each submatrix and predicted counts for overlapping submatrices were averaged. The resulting predicted high-coverage results were saved as a .hic file using Juicer tools (*26*), or as a .cool file using HiCPeaks Python implementation for downstream analysis.
