# Supplementary material for: HiC2Self: Self-supervised denoising for bulk and single-cell Hi-C contact maps
Source: Sci Adv. 2026 Jul 23;12(30):eadu8060. doi: 10.1126/sciadv.adu8060 (PMC13394478; doi:10.1126/sciadv.adu8060)
Supplement: Supplementary file 1 — Figs. S1 to S6 Tables S1 and S2 [file sciadv.adu8060_sm.pdf]

Science Advances
AAAS

Supplementary Materials for

HiC2Self: Self-supervised denoising for bulk and single-cell Hi-C contact maps

Rui Yang *et al*.

Corresponding author: Christina S. Leslie, lesliec@mskcc.org

Sci. Adv. **12**, eadu8060 (2026)
DOI: 10.1126/sciadv.adu8060

This PDF file includes:

Figs. S1 to S6
Tables S1 and S2

Fig. S1.

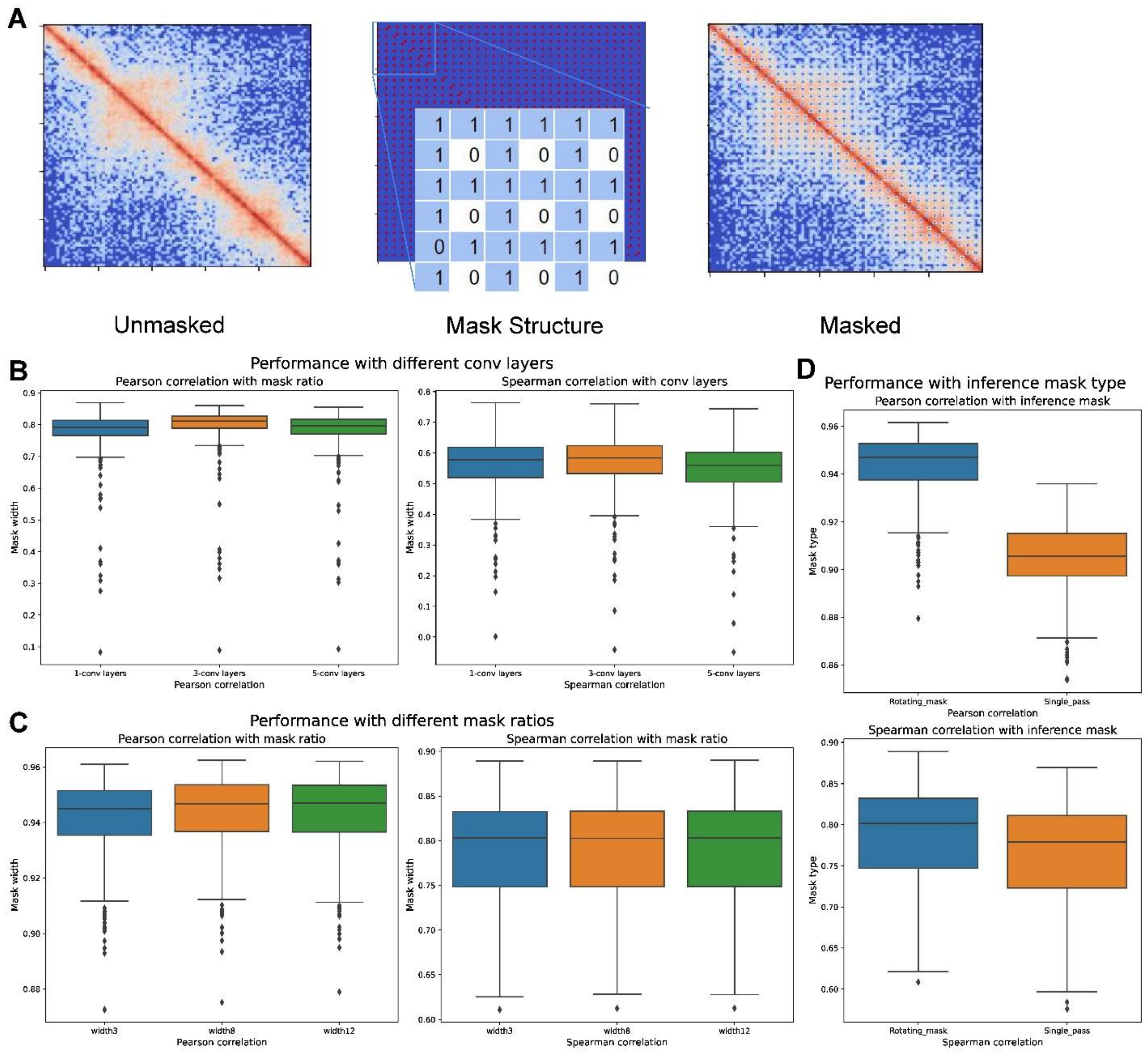

Figure S1: HiC2Self mask and model structure evaluations. (A) Mask structure of HiC2Self. (Left) Example low-coverage input matrix without a mask. (Middle) Diagram and a zoomed-in view of the mask structure. The mask is diagonally symmetric, where 0 entries are in the masked regions and 1 entries are in the unmasked regions. (Right) Same example input matrix multiplied by the mask. **(B)** HiC2Self structure analysis. Pearson and Spearman correlation of HiC2Self with 1 convolutional layer before prediction head (blue), 3 convolutional layers (model default, orange), and 5 convolutional layers (green). The model with 3 convolutional layers slightly outperformed the other two models. A two-sided Wilcoxon rank sum test of the correlation is shown as follows: (Pearson) 1-layer vs. 3-layer, p=3.91e-15; 3-layer vs. 5-layer, p=2.5e-10; 1-layer vs. 5-layer, p=0.094; (Spearman) 1-layer vs. 3-layer, p=0.098; 3-layer vs. 5-layer, p=2.3e-6; 1-layer vs. 5-layer, p=0.002. **(C)** HiC2Self mask structure exploration. HiC2Self with mask width=3 (one masked pixel for every 3 pixels, blue), width=8 (orange), and width=12 (green). HiC2Self showed

similar performance with mask ratio, with sparser masks slightly better. A two-sided Wilcoxon rank sum test of the Pearson and Spearman correlation is shown as follows: (Pearson) mask 3 vs. mask 8, p=0.018; mask 8 vs. mask 12, p=0.787; mask 3 vs. mask 12, p=0.008; (Spearman) mask 3 vs. mask 8, p=0.924; mask 8 vs. mask 12, p=0.958; mask 3 vs. mask 12, p=0.885. **(D)** HiC2Self inference with/without a mask. Pearson and Spearman correlation between the model inference with a rotating mask (default, blue) and a single-pass prediction without mask (orange). HiC2Self inference with a rotating mask provides more accurate predictions.

Fig. S2.

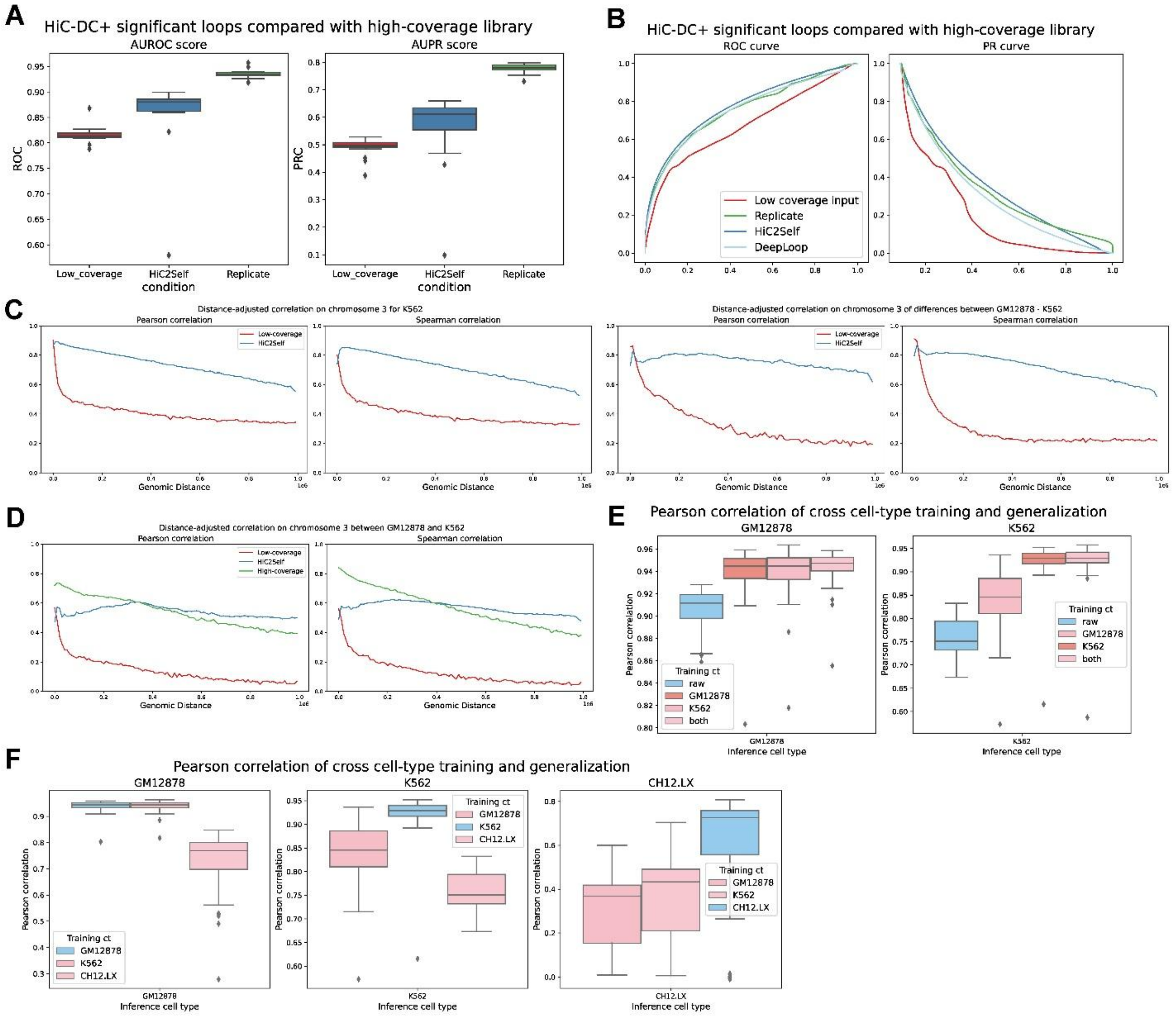

Figure S2: Evaluation of HiC2Self on bulk Hi-C data. (A) AUROC and AUPR of significant interactions. All libraries are bulk GM12878 binned at 10 kb resolution, and significant interactions are identified using HiC-DC+ (*17*). Red boxes show the AUROC and AUPR score of significant interactions comparing low-coverage vs. high-coverage libraries, across all chromosomes. Blue boxes show the performance of HiC2Self, and green boxes show the results of a deeply sequenced biological replicate Hi-C library. **(B)** ROC and PR curves for mid-range (<=2 Mb) cis-interactions at 5 kb resolution, comparing DeepLoop and HiC2Self. Significant interactions are identified by HiC-DC+ (*17*). The plot shows the performance of low-coverage data (red line, auROC=0.67, auPR=0.26), a deeply sequenced biological replicate (green line, auROC=0.75, auPR=0.39), DeepLoop trained with five replicates with sequencing depth ranging from 252.43 million to 1.78 billion reads (light blue, auROC=0.72, auPR=0.34), and HiC2Self trained with one low-coverage replicate with a sequencing depth of 202.10 million reads (dark blue, auROC=0.77, auPR=0.40). **(C)** Distance-adjusted correlations for cell-type specificity. (Left)

Distance-adjusted Pearson and Spearman correlation for chromosome 3 of K562, showing the correlation between low-coverage data vs. high-coverage data (red line) and HiC2Self recovery vs. high-coverage data (blue line). (Right) Distance-adjusted Pearson and Spearman correlation of the structural differences between GM12878 and K562. The difference is calculated by GM12878 - K562. **(D)** Distance-adjusted correlations for cell type similarity. The figure shows the Pearson and Spearman correlations between GM12878 and K562, calculated from high-coverage data (green), low-coverage data (red), and HiC2Self-recovered data (blue). **(E)** Pearson correlation of cross-cell-type training and generalization. (Left) HiC2Self's performance when trained on GM12878, K562, or both, evaluated on the GM12878 dataset. (Right) Performance for each training setting evaluated on the K562 dataset. **(F)** HiC2Self cross-cell-type training across species. (Left) HiC2Self's performance trained with GM12878, K562 or CH12.LX (mouse lymphoma cell line), evaluated on GM12878. (Middle) HiC2Self evaluated on K562. (Right) HiC2Self evaluated on CH12.LX.

Fig. S3.

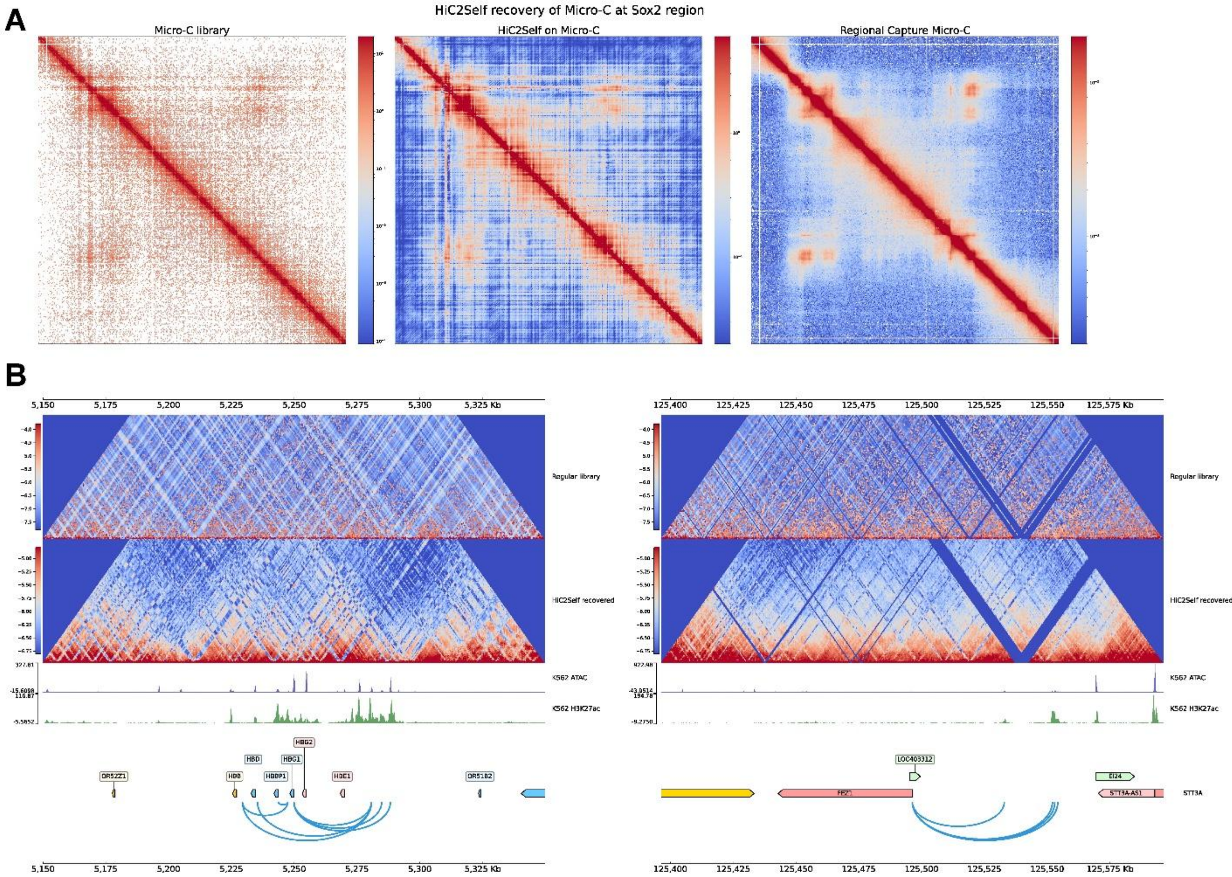

Figure S3: HiC2Self recovery at ultra-high resolution. (A) A visual comparison of HiC2Self recovery of mESC Micro-C data at 500 bp resolution around *Sox2* gene. The left panel shows the raw Micro-C contact map, the middle panel shows the HiC2Self recovery, and the right panel shows the RCMC map of the region. **(B)** HiC2Self reconstruction of K562 Hi-C library at 500 bp resolution, compared with the enhancer-promoter (E-P) interactions obtained from CRISPRi-FlowFISH data. (Left) E-P interaction region of *HBG1* gene. Top to bottom: raw Hi-C contact map at 500 bp resolution, HiC2Self recovered map, ATAC and K3K27ac tracks of the region, and E-P interaction identified from CRISPRi-FlowFISH. (Right) Comparison of the *FEZ1* gene region.

Fig. S4.

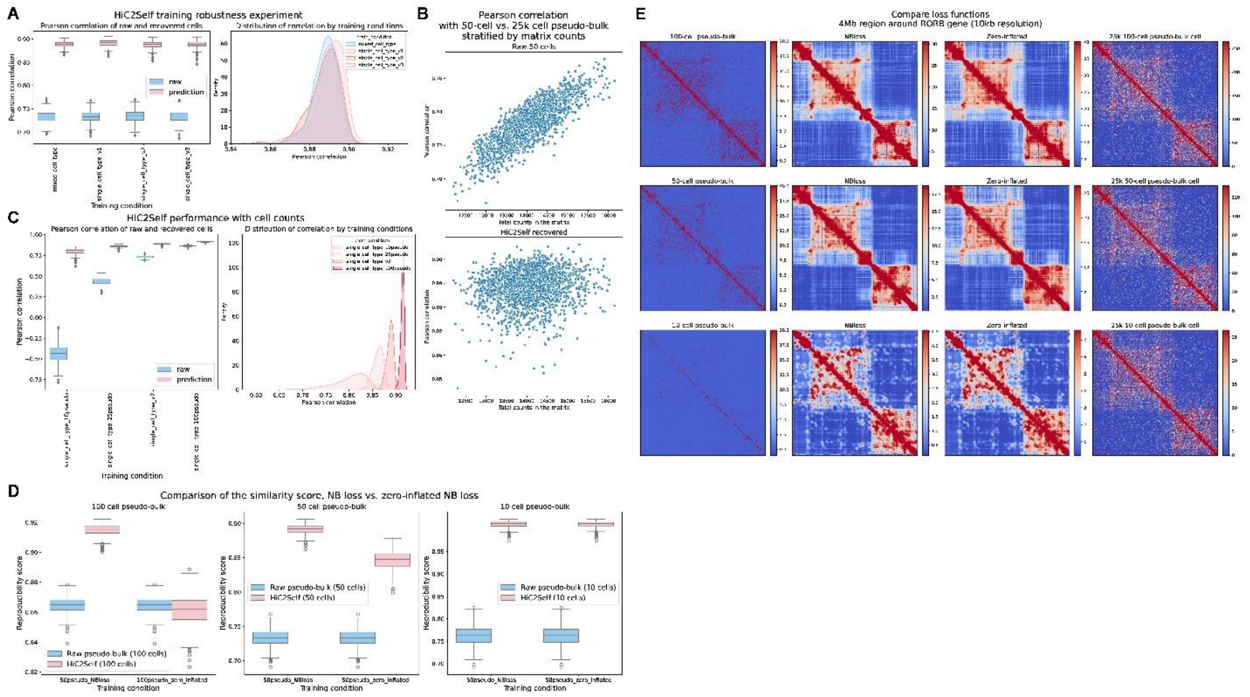

Figure S4: Robustness test of HiC2Self on single-cell application at pseudo-bulk level. (A) Reproducibility test of HiC2Self on pseudo-bulk cell selection. HiC2Self trained with a mixture of cell types is compared to training with a single cell type, 2T-RG-1. For the single-cell-type experiment, we resampled 400 pseudo-bulk samples, each composed of 50 cells as training data. We repeated the sampling and training 3 times to assess reproducibility. (Left) Pearson correlation between each resampled 50-cell pseudo-bulk sample and the pseudo-bulk of all cells from the cell type as the ground truth. Blue boxes show the correlations using raw pseudo-bulk cells, and pink boxes show correlations from HiC2Self-recovered cells. (Right) Distribution of Pearson correlation across all training runs, including those from multi-cell-type training and three single-cell-type runs. HiC2Self shows robust performance with respect to both training cell types and subsampling variability. **(B)** Read depth evaluation. Pearson correlation variation with read depth for pseudo-bulk samples composed of the same number of cells (50-cell pseudo-bulk). (Top) The relationship between read count and correlation for raw pseudo-bulk samples, where higher read depth results in better agreement with the 25K-cell pseudo-bulk ground truth. (Bottom) Same plot for HiC2Self recovered cells. After HiC2Self recovery, all samples achieve a correlation in the range of 0.85 to 0.95, showing that HiC2Self effectively reduces the variation dependent on read depth and enhances consistency. **(C)** Cell number evaluation. (Left) Pearson correlation of raw cells (blue) and HiC2Self recovered cells (HiC2Self) with 10-cell, 25-cell, 50-cell and 100-cell pseudo-bulk. All cells are from the 2T-RG-1 cell type and binned at 10kb resolution. Increasing the number of cells (read counts) in the raw data improves correlation with the ground truth, while HiC2Self consistently shows robust performance across all tested cell counts. (Right) Distribution of Pearson correlation of recovered cells to the ground truth. The higher cell number in the experiment, the better the performance, but all the experiments can achieve >0.75 Pearson correlations for almost all examples, indicating the model's robustness across a range of input

sizes. **(D)** Comparison of similarity-based reproducibility scores between HiC2Self models trained with a negative binomial (NB) loss and a zero-inflated negative binomial (ZINB) loss across 100-cell, 50-cell, and 10-cell pseudo-bulk data from the 2T-RG-1 cell type. Blue boxes show reproducibility of raw pseudo-bulk inputs, and pink boxes show reproducibility of HiC2Self-recovered maps. NB-based HiC2Self consistently shows slightly higher reproducibility scores than the ZINB-based model for 100-cell and 50-cell pseudo-bulk datasets, while the two losses perform comparably in the extremely sparse 10-cell setting. **(E)** Visual comparison of reconstructed contact maps using different loss functions for a 4 Mb region around the *RORB* gene at 10 kb resolution. For each pseudo-bulk size (top to bottom: 100-cell, 50-cell, and 10-cell), panels show the raw pseudo-bulk input, HiC2Self recovery using the negative binomial loss, HiC2Self recovery using the zero-inflated negative binomial loss, and the high-coverage 25K-cell pseudo-bulk reference. Both loss functions substantially enhance structural features relative to the raw input, with NB- and ZINB-based reconstructions showing similar visual quality across pseudo-bulk sizes.

Fig. S5.

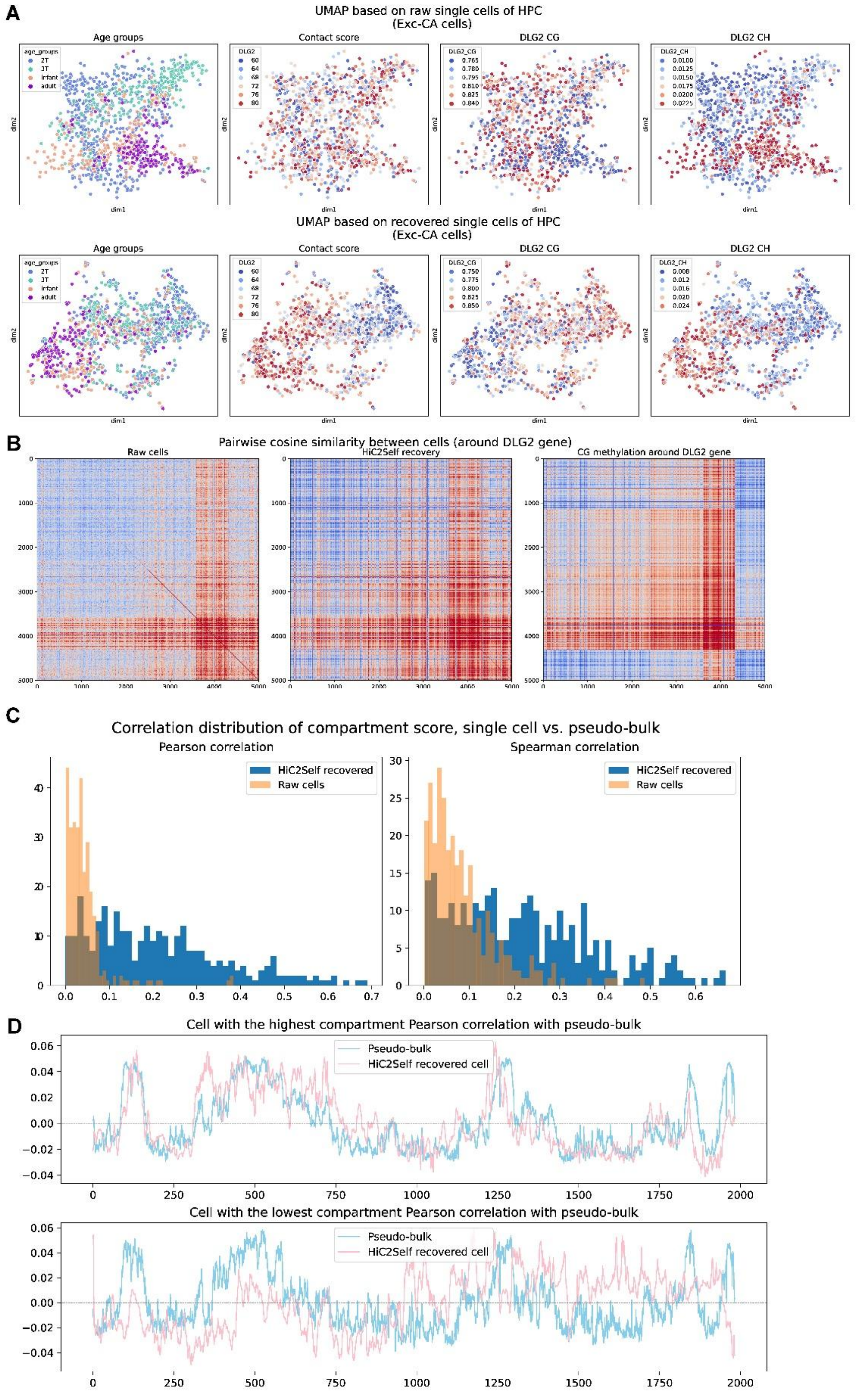
A
UMAP based on raw single cells of HPC
(Exc-CA cells)
Age groups
Contact score
DLG2 CG
DLG2 CH
UMAP based on recovered single cells of HPC
(Exc-CA cells)
B
Pairwise cosine similarity between cells (around DLG2 gene)
Raw cells
HiC2Self recovery
CG methylation around DLG2 gene
C
Correlation distribution of compartment score, single cell vs. pseudo-bulk
Pearson correlation
Spearman correlation
HiC2Self recovered
Raw cells
D
Cell with the highest compartment Pearson correlation with pseudo-bulk
Pseudo-bulk
HiC2Self recovered cell
Cell with the lowest compartment Pearson correlation with pseudo-bulk

Figure S5: UMAP clustering with local 3D structure of 20 Mb around *DLG2* gene. (A) All the cells are from the same cell type, Exc-CA. (Top) UMAP using the local 3D structure within 20 Mb around the *DLG2* gene from raw single cells. (Bottom) UMAP using the 20 Mb region local structure from HiC2Self recovered single-cell contact maps. **(B)** Heatmap of pairwise cosine similarity of normalized cells across all cell types around *DLG2* gene. The left panel shows the raw cells, the middle panel shows the HiC2Self recovered cells, and the right panel shows the CG methylation around the *DLG2* gene. The reproducibility score (*18*) of the cosine-similarity matrix is 0.83 between the raw cell matrix and the CG methylation similarity matrix, and 0.95 between HiC2Self recovery and CG methylation. HiC2Self recovery therefore shows more consistent correspondence of 3D structural patterns with CG methylation. **(C)** Distribution of Pearson (left) and Spearman (right) correlations between single-cell compartment (PC1) scores and the corresponding pseudo-bulk compartment scores for sampled single cells across five hippocampal progenitor cell (HPC) samples. Correlations are shown for raw single-cell contact maps (orange) and HiC2Self-recovered single-cell contact maps (blue). HiC2Self shows improved agreement with pseudo-bulk compartment profiles after HiC2Self recovery. **(D)** Representative examples of compartment scores along chromosome 3 for individual single cells. For each example, the compartment score calculated from the HiC2Self-recovered single-cell contact map (pink) is shown alongside the pseudo-bulk compartment score for the corresponding sample (blue). The top panel shows a single cell with high Pearson correlation (r=0.689) to the pseudo-bulk reference, while the bottom panel shows a single cell with low Pearson correlation (r=0.008). HiC2Self-recovered single-cell compartment profiles remain consistent with the pseudo-bulk reference across a range of correlation strengths.

Fig. S6.

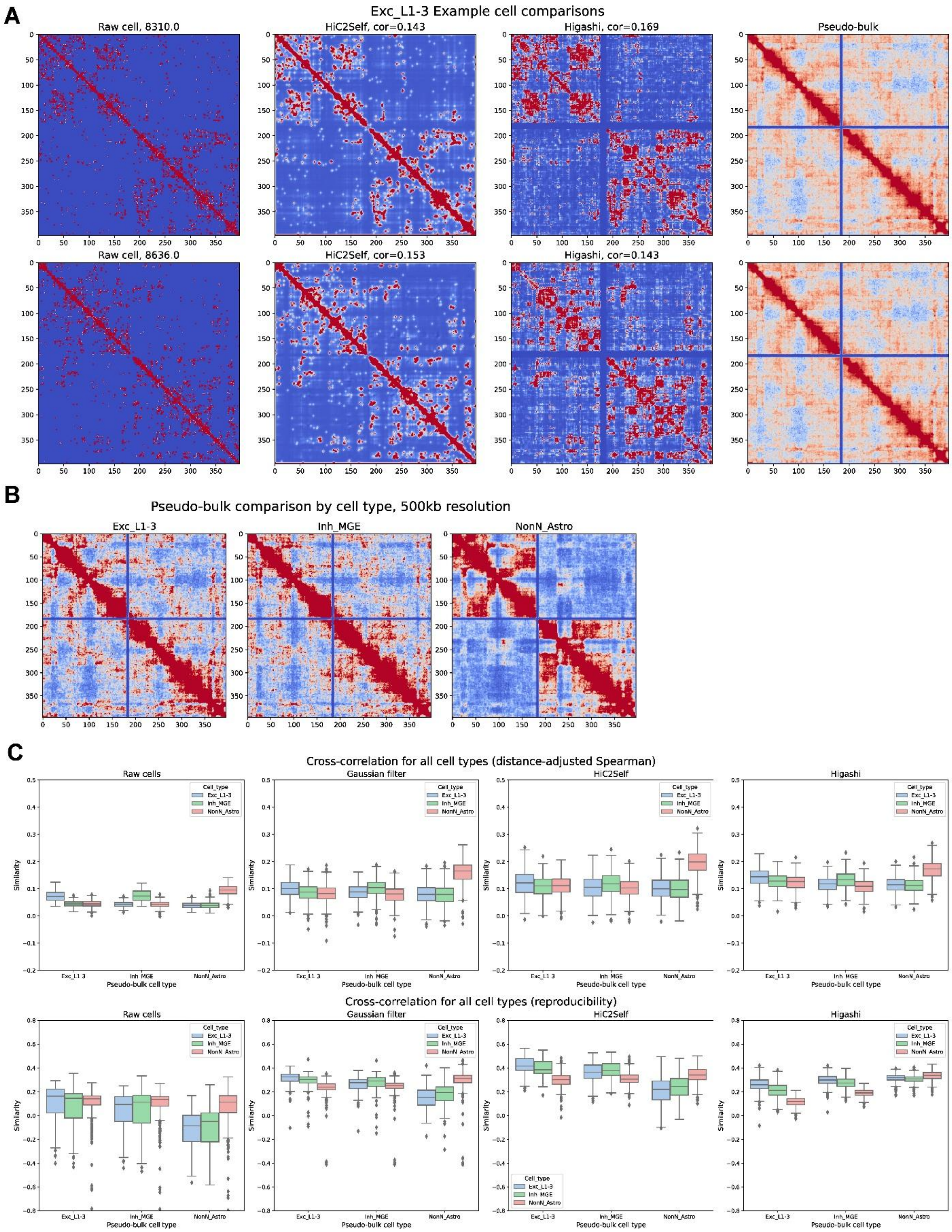

A
Exc_L1-3 Example cell comparisons
Raw cell, 8310.0
HiC2Self, cor=0.143
Higashi, cor=0.169
Pseudo-bulk
Raw cell, 8636.0
HiC2Self, cor=0.153
Higashi, cor=0.143
B
Pseudo-bulk comparison by cell type, 500kb resolution
Exc_L1-3
Inh_MGE
NonN_Astro
C
Cross-correlation for all cell types (distance-adjusted Spearman)
Raw cells
Gaussian filter
HiC2Self
Higashi
Cell_type
Exc_L1-3
Inh_MGE
NonN_Astro
Similarity
Pseudo-bulk cell type
Cross-correlation for all cell types (reproducibility)

Figure S6: Benchmark with Higashi at 500 kb resolution. (A) Two random example cells from the Exc_L1-3 cell type, showing left to right: raw single-cell contact map, HiC2Self recovery, Higashi recovery, pseudobulk contact map for this cell type. The top row shows an example cell where the distance-adjusted Spearman correlation is higher in Higashi, and the bottom row shows an example cell where the correlation is higher is HiC2Self. **(B)** A visual comparison of the pseudobulk cells from three distinct cell types (Exc_L1-3, Inh_MGE and NonN_Astro). **(c)** Cross-comparison of cells from these three distinct cell types (Exc_L1-3, Inh_MGE and NonN_Astro) compared with the corresponding pseudobulk map. Left to right: comparison of raw single-cell maps, Gaussian smoothed cells, HiC2Self recovery and Higashi recovery. The top row shows the distance-adjusted Spearman correlation compared with the pseudo-bulk, and the bottom row shows the reproducibility score.

Table S1. Model scalability with input size.

Matrix Size	Time per Epoch (sec)		Memory of GPU Peak (GB)		Memory of CPU (GB)	
	Mean	Std	Mean	Std	Mean	Std
**200**	30.14	0.13	0.06	0.0	1.37	0.01
**400**	97.88	0.32	0.21	0.0	2.14	0.10
**800**	805.92	0.60	0.80	0.0	4.93	0.10
**1000**	1237.83	7.24	1.26	0.0	7.06	0.11

Table S2. Model scalability with mask ratio.

Mask Ratio	Time per Training Epoch (sec)		Time per 100 Samples during Inference (min)
	Mean	Std	Mean (400×400 matrices)
**3**	4742.40	3.40	2.69
**8**	1507.53	1.24	4.35
**12**	1213.80	1.34	8.30
